# Long‐term longitudinal changes in axial length in the Caucasian myopic and hyperopic population with a phakic intraocular lens

**DOI:** 10.1111/aos.14647

**Published:** 2020-10-29

**Authors:** Zoraida S. Gaurisankar, Gwyneth A. van Rijn, Geert W. Haasnoot, Virginie J. M. Verhoeven, Caroline C. W. Klaver, Gregorius P. M. Luyten, Jan‐Willem M. Beenakker

**Affiliations:** ^1^ Department of Ophthalmology Leiden University Medical Center Leiden The Netherlands; ^2^ Department of Immunohematology and Blood Transfusion Leiden University Medical Center Leiden The Netherlands; ^3^ Department of Ophthalmology Erasmus University Medical Center Rotterdam The Netherlands; ^4^ Department of Clinical Genetics Erasmus University Medical Center Rotterdam The Netherlands; ^5^ Department of Epidemiology Erasmus University Medical Center Rotterdam The Netherlands; ^6^ Department of Ophthalmology Radboud University Medical Center Nijmegen The Netherlands; ^7^ Department of Radiology C.J. Gorter Center for High‐Field MRI Leiden University Medical Center Leiden The Netherlands

**Keywords:** axial elongation, axial length, hyperopia, myopia, phakic intraocular lens, refractive error

## Abstract

**Purpose:**

To determine the long‐term longitudinal axial length changes in myopic and hyperopic adults with an iris‐fixated phakic intraocular lens (pIOL).

**Methods:**

The medical records of patients aged ≥18 years with myopia or hyperopia who were treated with pIOL implantation between 1996 and 2011 for refractive correction with a minimum follow‐up of 5 years after pIOL implantation were analyzed. The main outcome measure was change in ocular axial length over time.

**Results:**

149 eyes of 149 myopic patients and 27 hyperopic eyes of 27 patients were included in this study. Mean patient age was 37.1 ± 10.4 years (35% male) in the myopic group and 39.4 ± 9.4 years (4% male) in the hyperopic group. The eyes of the myopic patients showed a significant mean increase in axial length of 0.45 ± 0.61 mm after a mean follow‐up time of 144 ± 38 months (p < 0.001). In 26 eyes (17%), the axial length had increased by ≥1 mm. The mean annual axial length increase was 0.04 ± 0.06 mm. Axial elongation was associated with a higher degree of myopia (p < 0.001) and younger age (p = 0.02). The eyes of the hyperopic patients showed no change in axial length over time.

**Conclusions:**

Myopic eyes corrected with an iris‐fixated pIOL show continuous increase in axial length at an adult age. Although this study is limited to subjects with a pIOL, this is the first time myopization in Caucasian adults has been reported in a large long‐term longitudinal study.

## Introduction

Ocular axial length is the most important biometric value determining refractive errors. During the process of emmetropization in infancy, the axial length increases in line with the focal length of the eye’s optics until it reaches the adult axial length at the age of 13 years and is thought to remain stable thereafter (Larsen, 1971; Flitcroft, 2014). It was not until the end of the nineteenth century that myopic shifts in adults were firstly described, but these refractive changes appear to primarily reflect changes in the optical power of the lens rather than in axial length (Flitcroft, 2014). Adults without cataract and with myopia, especially high myopia (at least −6 dioptres), may show myopic progression, ultimately carrying risks of serious vision‐threatening complications (McBrien & Adams, 1997; Saka et al., 2010; Medina, 2015). Given the increasing prevalence of myopia worldwide (Wu et al., 2019), a better understanding of the progression might help its prevention.

Ocular axial elongation has been reported in a few long‐term longitudinal studies. In myopic Asian adults, the yearly increase ranged from 0.04 to 0.30 mm with mean follow‐up periods varying from 2 to 8 years (Saka et al., 2010; Ohsugi et al., 2017; Torii et al., 2017). In Asian countries, (high) myopia is more prevalent than in Caucasian countries (Holden et al., 2016; Wong & Saw, 2016), but the ethnical differences in axial length progression are unknown. It might therefore be inaccurate to extrapolate the longitudinal findings on axial length progression to myopic adults in Caucasian countries or other regions outside of Asia. No longitudinal literature is available on axial length in adults with hyperopia.

High ametropia can successfully be corrected with phakic intraocular lens (pIOL) implantation. In our clinics, implantation of the phakic iris‐fixating Artisan lens (Ophtec BV, Groningen, The Netherlands) has been performed since 1996.

The main purpose of this study was to assess long‐term longitudinal axial length changes in myopes and hyperopes after phakic Artisan lens implantation. To our knowledge, this is the first study that solely focuses on long‐term longitudinal data on axial length changes in Caucasian adults.

## Materials and Methods

### Case selection

This longitudinal observational study adhered to the tenets of the Declaration of Helsinki and was approved by the medical ethical committee of the Leiden University Medical Center (LUMC). Informed consent was obtained from all patients. Medical records from 1996 to 2018 were searched at our clinics for patients with a history of pIOL implantation for refractive correction of myopia or hyperopia and a follow‐up time of ≥5 years after surgery. All surgeries were performed by one surgeon (GL) in two different clinics: Erasmus Medical Center, Rotterdam, and LUMC, Leiden. If the patient had undergone pIOL implantation in both eyes, only one eye was randomly selected and included. If the medical history showed a second operation during follow‐up, such as a cataract extraction, only the last data before the second surgery were used. The eyes were divided into two groups: myopes (patients corrected with Artisan Myopia Model 204 or 206 pIOL) and hyperopes (patients corrected with Artisan Hyperopia model 203 pIOL).

### Medical record review

Detailed medical history was reviewed to gather information on the following: the axial length (AL) obtained by one experienced examiner with the immersion A‐scan using the mean of three measurements (Alcon Biophysic OcuScan, Version 3.02; Fort Worth, TX, USA), Lenstar LS 900 (Haag‐Streit AG, Koeniz, Switzerland) or IOLMaster (Carl Zeiss Meditec AG, Jena, Germany); spherical equivalent (SE); keratometry measured by automated keratometry (the average of dioptric power of the steepest and flattest meridian, *K*_avg_, was calculated for analysis) using the Topcon RM‐A2000 (Tokyo Optical Co., Tokyo, Japan) or Topcon KR8900 Ref (Tokyo Optical Co., Tokyo, Japan); central corneal thickness (CCT); and anterior chamber depth (ACD) measured by immersion A‐scan and after 2003 by Pentacam (Oculus Optikgeräte GmbH, Wetzlar, Germany). Measurements were recorded at the first preoperative visit and the last follow‐up visit. For SE, in addition to the preoperative and the last measurement, also 3 months postoperative records were recorded and used for comparison with last visit SE. Furthermore, when present, fundus photographs taken at baseline were collected to record the presence or absence of posterior staphyloma in myopes. Using the International Photographic Classification and Grading System for Myopic Maculopathy (Ohno‐Matsui et al., 2015), the photograph was carefully screened on features of posterior staphyloma by one examiner and either scored ‘present’ or ‘absent’.

The primary end‐point was the change in AL over time. The secondary end‐point was to identify predictors of possible AL changes.

### Statistical analysis

The myopic and the hyperopic study groups were analyzed separately with ibm spss Statistics version 25 for Windows (SPSS Inc., Chicago, IL, USA). Descriptive statistics, including means, standard deviations, proportions and frequency distributions, were generated for subject characteristics. Scatter plots and box plots were used to visualize the data. The change of AL over time in each eye was analysed by examining the difference between preoperative and last visit AL measurement for statistical significance using the Wilcoxon signed‐rank test. In the majority of the study eyes, different biometry devices were used over time to obtain preoperative and last visit AL. To assess whether the use of different biometry devices affected the AL measurements, the nonparametric Kruskal–Wallis test (due to the limited sample size in the different groups) was performed to compare each combination of devices.

Univariate and multivariate regression analyses were used to assess possible predictors of AL changes including age, sex, right/left eye, SE at baseline, AL at baseline, *K*_avg_, ACD, CCT and the presence of a staphyloma posterior. In addition, to compare AL change in myopic eyes with and without staphyloma posterior, an independent *t*‐test was used.

The change in SE over time was analysed with a paired *t*‐test (SE 3 months postoperative versus SE at last visit). p < 0.05 was considered statistically significant.

## Results

### Patient characteristics

Table 1 shows demographic and clinical features of the study subjects in the two study groups. The myopic group included 149 eyes of 149 patients, and the hyperopic group, 27 eyes of 27 patients. The mean age was 37.1 ± 10.4 years and 39.4 ± 9.5 years in the myopic and hyperopic groups, respectively. The mean AL at the first visit was 28.06 ± 2.20 and 21.19 ± 0.81 mm, and the mean follow‐up time was 144 ± 38 and 146 ± 41 months, respectively. There were no significant differences in sex, age or postoperative SE or mean follow‐up time between the two groups.

**Table 1:** Patient characteristics.

Variable	Myopic group	Hyperopic group	p‐Value
Eyes (count)	149 (77 right, 72 left)	27 (13 right, 14 left)	
Sex (male:female, %)	35:65	48:52	0.109
Mean age at first visit ± SD (min–max, years)	37.1 ± 10.4 (18 to 58)	39.4 ± 9.4 (18 to 56)	0.561
Mean SE at first visit ± SD (min–max, D)	−12.26 ± 4.87 (−2.75 to −32.50)	+6.63 ± 1.77 (+1.75 to +10.50)	<0.001
Mean SE 3 months after pIOL implantation ± SD (min–max, D)	−0.28 ± 0.83 (−6.63 to +1.00)	−0.11 ± 0.57 (−1.13 to +1.50)	0.994
Mean AL at first visit (min–max, mm)	28.06 ± 2.20 (24.80 to 37.27)	21.18 ± 0.81 (19.71 to 22.76)	<0.001
Mean follow‐up time ± SD (min–max, months)	144 ± 38 (56 to 243)	146 ± 41 (75 to 238)	0.680

AL = axial length, D = dioptres, max = maximum, min = minimum, mm = millimetres, SD = standard deviation, SE = spherical equivalent.

### Axial length

**Fig. 1:**
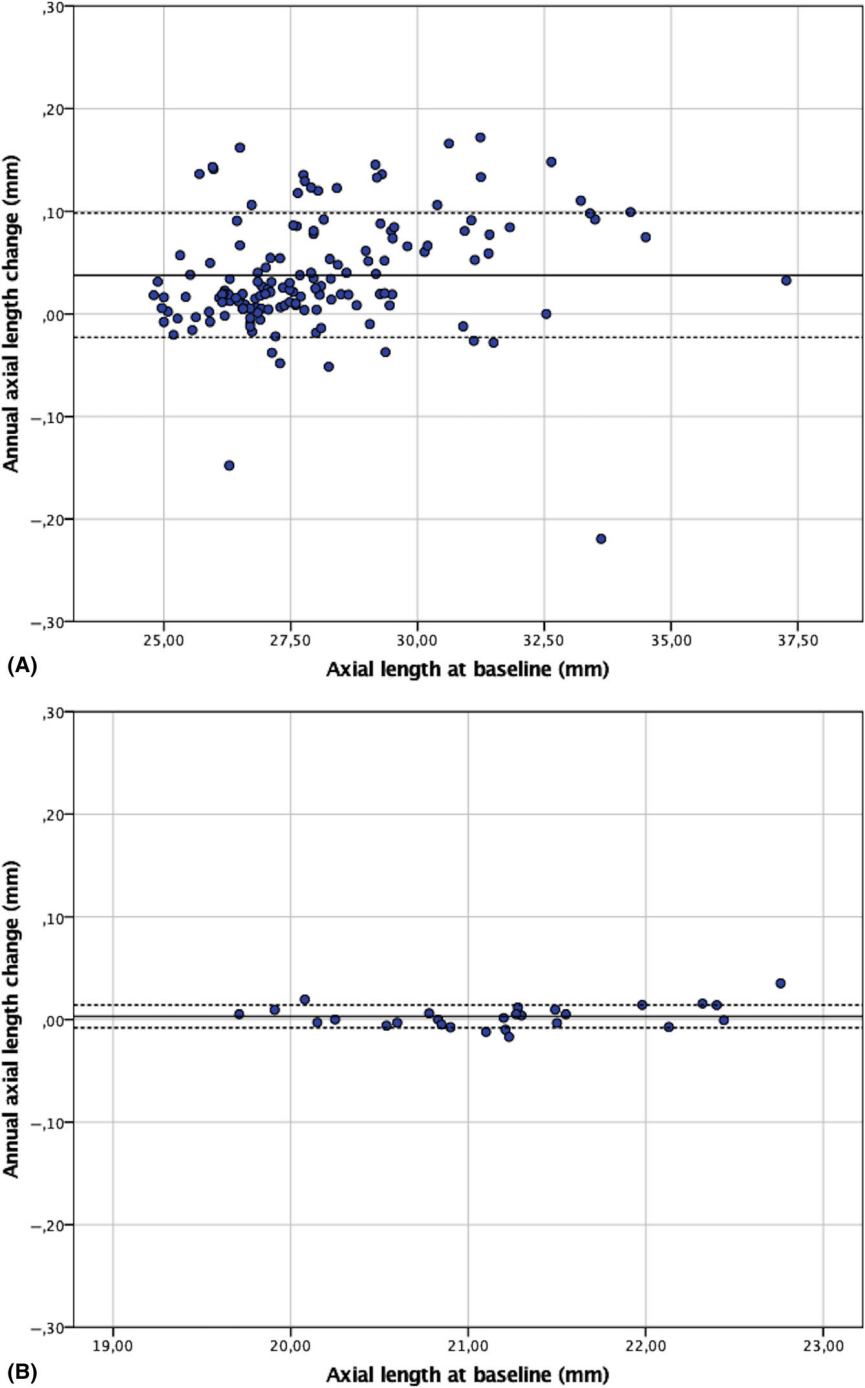
Scatter plot showing differences in axial length (AL) changes over time in (A) myopic eyes and (B) hyperopic eyes with a pIOL in millimetres (mm). A positive difference responds to an increase in AL between the first and last measurements. *Black line*: mean. *Black dashed lines*: 95% confidence interval of the limits of agreement.

In the myopic group, a significant difference was found between the first and last visit AL measurements of 0.45 ± 0.61 after a mean follow‐up time of 144 ± 38 months (p < 0.001). In 26 myopic eyes (17.4%), the AL had increased by ≥1 mm. The annual AL change was 0.038 ± 0.055 mm, inferring a 0.38 mm AL increase over a 10‐year time span. In the hyperopic group, no significant difference was found between the first and last AL measurements (p = 0.231). As shown in the scatter plot in Fig. 1, the AL increases over time in myopic eyes with a pIOL (A) and is stable in the hyperopic eyes with a pIOL (B).

Different devices were used for the AL measurements made preoperatively and at final visit because of the more recent introduction of optical biometry. More than 90% of the preoperative AL measurements were obtained with the A‐scan in both groups, in contrast to the AL measurements at the last visit, which were obtained with the Lenstar or IOLMaster in 95% of the cases in the myopic group and in all of the cases in the hyperopic group. The Kruskal–Wallis test revealed no difference in AL change among the different combinations of device used for preoperative and final measurement in the myopic (chi‐square = 8.25, p = 0.083, df = 4) and hyperopic group (chi‐square = 3.10, p = 0.213, df = 2), as shown in Fig. S1.

### Risk factors for AL progression in myopia

**Fig. 2:**
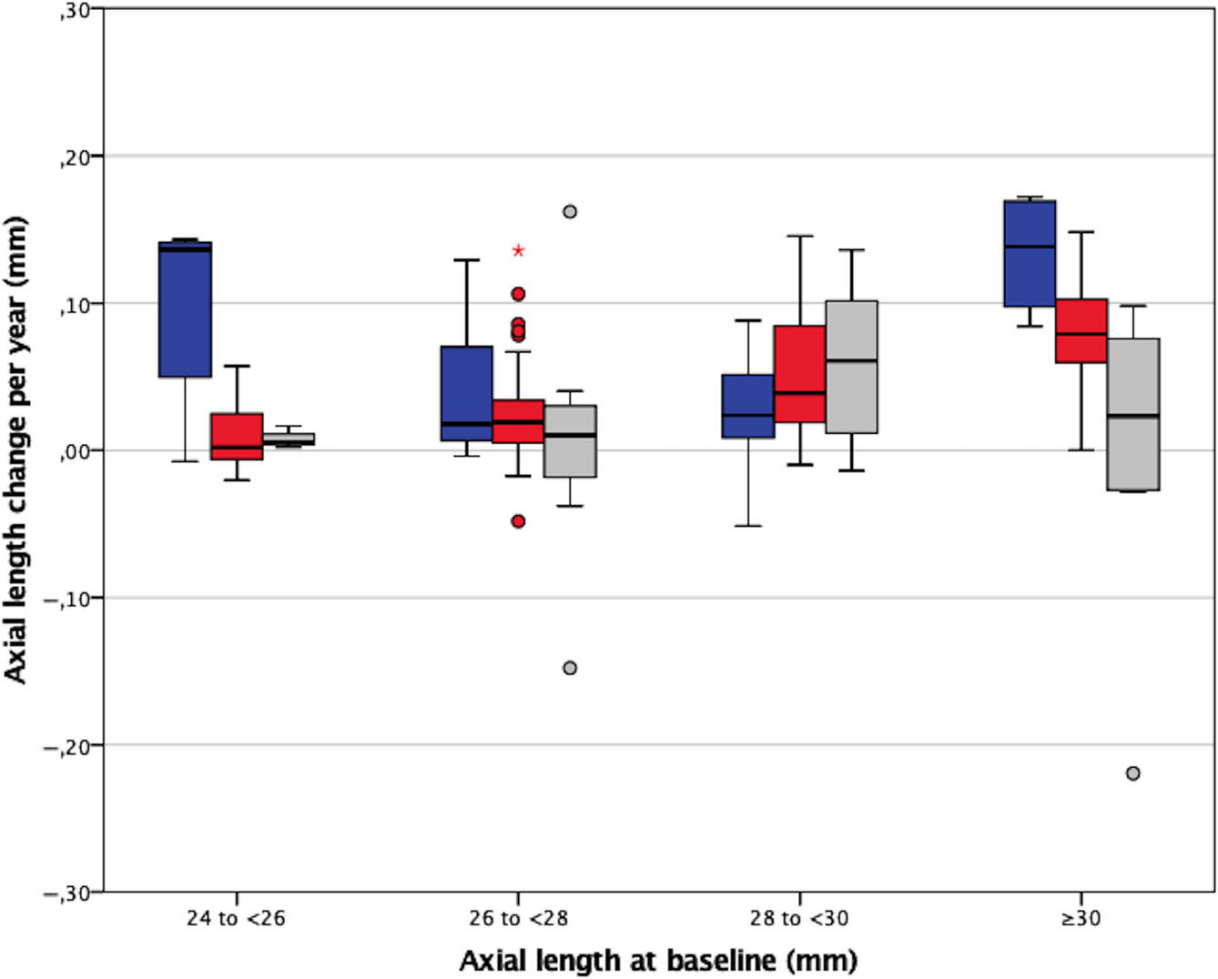
Box plot showing axial length (AL) change per year in millimetres (mm) associated with axial length at baseline with respect to age at baseline. *blue*: 18–30 years, *red*: 31–45 years, *grey*: 46–60 years.

In order to identify the risk factors for AL change in myopic eyes with an pIOL, univariate and multivariate regression analyses were used to examine baseline variables affecting AL including age, sex, right/left eye, SE at baseline, AL at baseline, *K*_avg_, ACD, CCT and the presence of a staphyloma posterior (Table 2). SE at baseline was found to be the most important risk factor for AL progression: eyes with higher degrees of myopia were associated with accelerated AL progression. A younger age was also a significant risk factor, although the effect on AL change was only small. The other factors were not found to be predictors for AL change. Figure 2 shows a box plot of AL change per year for different age groups and axial length at baseline. The largest increase in AL over time is seen in the youngest group (16–30 years of age) with an AL of ≥30 mm at baseline.

**Table 2:** Univariate and multivariate analyses for axial length (AL) change in myopic eyes with a phakic intraocular lens.

Variables	Univariate analysis			Multivariate analysis		
	β coefficient	95% CI	p‐Value	β coefficient	95% CI	p‐Value
Age (years)	−0.001	(−0.002; 0.000)	**<0.001**	−0.001	(−0.002; 0.000)	**0.024**
Female sex	0.010	(−0.009; 0.029)	0.290			
SE	−0.004	(−0.005; −0.002)	**<0.001**	−0.003	(−0.005; −0.001)	**<0.001**
AL at baseline	0.007	(0.004; 0.010)	**<0.001**			
*K*_avg_ (D)	0.002	(−0.003; 0.008)	0.577			
ACD (mm)	0.009	(−0.016; 0.034)	0.482			
CCT (µm)	0.000	(0.000; 0.001)	**0.039**	0.000	(0.000; 0.001)	**0.060**
Posterior staphyloma*	0.023	(0.003; 0.042)	**0.024**			

95% CI = 95% confidence interval, ACD = anterior chamber depth, CCT = central corneal thickness, D = dioptres, *K*_avg_ = average keratometry, mm = millimetres, SE = spherical equivalent, µm = micrometres. Bold values denote statistical significance at the p < 0.05 level.

* Univariate analysis performed on 158 of 296 myopic eyes with a preoperative fundus photograph.

In 81 of 149 myopic eyes preoperative fundus photographs had been taken. Of these 81 eyes, 43% had a posterior staphyloma and 57% had a normal fundus. Univariate analysis showed posterior staphyloma to be a significant risk factor for AL progression (p = 0.024), but this variable was not included in the multivariate analysis because of a possible selection bias. An independent samples *t*‐test showed no difference of AL change in the eyes with a staphyloma versus eyes without a staphyloma (p = 0.129).

### AL progression and change in SE

Myopic eyes showed a significant difference in SE of −0.24 ± 0.90 D (p < 0.001) between the first and second measurements over the mean follow‐up of 144 ± 38 months using a paired t‐test. The mean annual SE change was a myopization of 0.07 ± 0.12 D per year and showed a moderate correlation with the annual AL increase (p < 0.001, Pearson’s coefficient: −0.334). There was no significant change in SE in the hyperopic group.

## Discussion

In this study, we investigated longitudinal changes in AL in adults with myopia and hyperopia, with an iris‐fixated pIOL, during a mean follow‐up time of 12 years. The myopic eyes were found to have significant AL elongation over time with a mean increase of 0.38 mm/10 years, but eyes with higher myopic errors tend to grow even more. The AL in hyperopic eyes did not change over time.

In literature, a few longitudinal studies on AL progression have been performed in myopic Asian adults: Saka et al. (2010) found a median increase in AL per year of 0.08 mm (range, −0.16 to 0.43 mm/year) using A‐scan ultrasonography in high myopes during a mean follow‐up period of 8.2 years in 184 eyes, which is twice the increase we found in our study population. Similar to our findings, no difference in AL change was found between eyes with posterior staphyloma (58%) and those without. Using IOLMaster, Torii et al. (2017) reported a similar rate of AL elongation of 0.38 mm/5 years in highly myopic adult patients without staphyloma posterior after pIOL implantation. Recently, Chen et al. (2019) presented an even more dramatic increase in AL of 0.30 mm/year in Chinese adults with high myopia during a follow‐up period of 5.4 years. However, the study group consisted of only 12 eyes of seven patients with a higher degree of myopia (mean of −16.4 D), compared to our study population. Ohsugi et al. (2017) reported significant axial elongation in myopic eyes with and without macular complications by using the IOLMaster. They examined four different groups: the nonhighly myopic group, the group with no complications, the myopic traction maculopathy group and the myopic choroidal neovascularization (CNV) group, with the yearly AL changes of 0.007 ± 0.02, 0.041 ± 0.05, 0.040 ± 0.05 and 0.081 ± 0.04 mm, respectively. In this study, the rate of AL increase in the high myopic groups without CNV is similar to our myopic study population, although our patients were younger. In Caucasians, long‐term longitudinal AL change was only earlier described by Jonker et al. (2019) in a subset of Dutch patients after pIOL implantation. With a follow‐up of up to 10 years, they reported an AL change of 0.11 mm/year in a subset of 24 eyes using optical biometry. The reason the previous studies reported more AL change is not clear. Our longer follow‐up, bigger sample size and ethnical differences may have influenced the results.

The study of Ohsugi et al. (2017) also mentioned the most important implication of AL progression in myopic adults, namely the development of visual impairment as a result of pathologic myopia. In particular, CNV eyes showed greater increases, indicating that larger changes in AL may require careful follow‐up (Ohsugi et al., 2017). Also, in the myopic European population, longer AL is associated with visual impairment (Tideman et al., 2016). In our myopic study population, 17% of the eyes show an AL increase of ≥1 mm over time, and the most accelerated AL increase appears to occur in adults of 18–30 years with a baseline AL of ≥30 mm (Fig. 2). These individuals who do not have myopic complications yet may have the greatest risk of developing visual impairment and may benefit from possible preventive therapies in future. With regard to the effect on refraction, the change in SE over time of −0.07 D we found in our study is smaller than average relation of −2.0 to −2.5 D per mm change in axial length, which can be attributed to the relatively long eyes in our myopic population (Meng et al., 2011; Cruickshank & Logan, 2018).

In addition, our findings of accelerated AL change in younger adults raise the question in what manner the rate of progression develops over time in highly myopic eyes. The 5‐year AL changes, documented in the previously mentioned Asian studies (Saka et al., 2010; Torii et al., 2017; Chen et al., 2019), may suggest that a great part of AL increase occurs at a younger age. In our study, follow‐up time (ranging from 5 up to 20 years) was not found to be a significant risk factor for AL elongation, which further supports this hypothesis. To explore the exact process of AL progression over time, additional research on longitudinal AL changes measured at several time‐points is indicated.

Although this study is performed in Caucasian eyes with a pIOL, it is expected that these results can be extrapolated to the general Caucasian myopic and hyperopic population without pIOL. The results of our study show a stable AL in hyperopic patients with a similar pIOL, and there is no known effect of Artisan pIOL implantation on the process of axial elongation from literature. Previous studies showed that changes in AL measurement before and after pIOL by A‐scan are insignificant (Shin et al., 2013), while measurements by IOLMaster are described to be longer postoperative Artisan pIOL implantation with differences of 0.03 (Lee et al., 2018) to 0.12 mm (Shin et al., 2013). However, the latter findings cannot explain the much greater difference of 0.45 mm found in our study.

In myopic patients corrected with laser refractive surgery, such as laser‐assisted in situ keratomileusis (LASIK) and photorefractive keratectomy (PRK), myopic regression has widely been observed (Wagoner et al., 2011; Lin et al., 2012; Alio et al., 2015; Pokroy et al., 2016) and seems to increase with higher corrections (Pokroy et al., 2016). Multiple factors may lead to myopic regression in these patients such as epithelial hyperplasia, changes in the biomechanical properties of the cornea and the increase in central corneal power (Chayet et al., 1998; Alió et al., 2008). Additionally, an increase in AL in these eyes may partially explain the myopic regression. More longitudinal studies are needed to examine this hypothesis.

In the univariate analysis, besides the degree of myopia and younger age, the presence of a staphyloma posterior was found to be a predictor of AL increase, though the extent of AL elongation did not differ between eyes without staphyloma and eyes with staphyloma. In the latter, especially in higher degrees of myopia, the sclera is often thinner than usual (McBrien & Gentle, 2003) and one might expect more elongation. Further in‐depth studies are needed on the relation between posterior staphyloma and AL change, preferably using three‐dimensional MRI or B‐scan for more accurate identification of (types of) posterior staphyloma.

The exact aetiology of myopia progression in adults is not well understood. McBrien (McBrien & Gentle, 2003; McBrien, 2013) states that there is an important role for the sclera in the development of myopia and progression towards pathologic myopia and the development of posterior staphyloma. These changes are probably caused by both nature and nurture. Genome‐wide association studies have provided evidence of genetic predisposition of AL and refraction (Cheng et al., 2013). In addition, environmental and behavioural factors, such as urbanization, education, socio‐economic status and near work, which have well‐established links with AL elongation in children (Williams et al., 2015; Zhou et al., 2017), may play a role in further elongation later in life.

Nevertheless, the fact that different biometers were used for the first and last AL measurements is a limitation of this study. This was unavoidable because the first data were collected more than 10 years ago when ultrasonography was the only clinical method available to measure AL. In our study, three different biometers were unavoidably used to measure AL throughout time. Though it is a limitation of our study, statistical analysis revealed no significant effect on our data in the myopic and hyperopic group. The fact that hyperopic eyes did not show AL changes further supports that the AL changes in myopic eyes cannot be explained by the use of different biometry devices. The IOLMaster and Lenstar strongly concur in measuring AL (Huang et al., 2009). Although AL measurements made with the immersion A‐scan also generally concur with those of the IOLMaster and Lenstar (Packer et al., 2002; Huang et al., 2017), there are some studies describing a difference of −0.11 to −0.25 mm (Montes‐Mico et al., 2011; Naicker et al., 2015; Wang et al., 2016). Although this small difference might explain the negative AL changes in the myopic and hyperopic eyes (Fig. 1), possibly caused by a misalignment of the A‐scan probe, it is too small to explain the overall change in AL in the myopic group. This is further confirmed by the lack of any statistically significant change in AL in the hyperopic eyes. It is also noteworthy that the preoperative AL measurement was excluded from the multivariate analysis because of its high correlation with SE (Pearson’s correlation coefficient = −0.940 with p < 0.001).

In conclusion, Caucasian patients with myopia, corrected with an iris‐fixated pIOL, show continuous AL elongation at an adult age. In 17% of the patients, ocular axial length growth was more than 1 mm over a mean time span of 12 years. The most important risk factor for AL progression is a higher degree of myopia, but also younger age was found to be a risk factor. The AL in hyperopic adults, corrected with an iris‐fixated pIOL, remains stable over time. Despite the fact that all patients were corrected with a pIOL, we assume myopic eyes in general may elongate in the same manner.

## Supporting information

**Fig. S1.** Box plot of axial length change for the different combinations of biometry device used for preoperative and final measurement in (A) myopic eyes and (B) hyperopic eyes.
