# Supplementary figures and images for: Long‐term longitudinal changes in axial length in the Caucasian myopic and hyperopic population with a phakic intraocular lens
Source: Acta Ophthalmol. 2020 Oct 29;99(4):e562–8. doi: 10.1111/aos.14647 (PMC8359445; doi:10.1111/aos.14647)

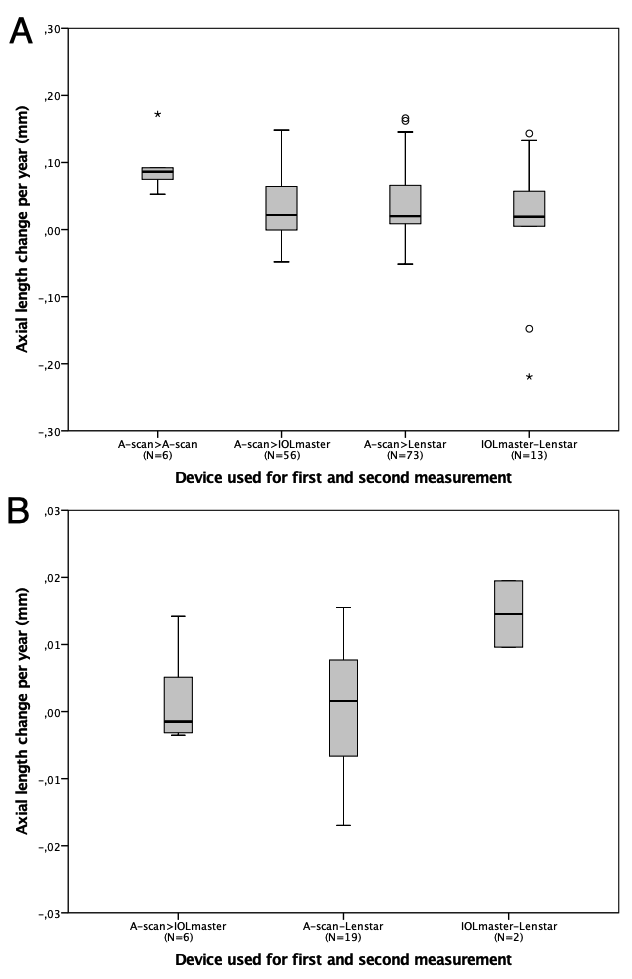

Supplement: Supplementary file 1 — Fig. S1. Box plot of axial length change for the different combinations of biometry device used for preoperative and final measurement in (A) myopic eyes and (B) hyperopic eyes. [file AOS-99-e562-s001.tif]
